# Composite resin color stability and surface roughness after exposure to beverages

**DOI:** 10.6026/973206300220749

**Published:** 2026-02-28

**Authors:** Ashish Kalawat, Kondaveeti Viswaja, Mukti Kansal, Omm Ritish Kumar, Anurag Aggarwal, Ahmad S Albahoth

**Affiliations:** 1 Department of Orthodontics and Dentofacial Orthopaedics, Maharaja Ganga Singh Dental College and Research Centre, Sri Ganganagar, Rajasthan, India; 2 Department of General Pathology, SRM Dental College, Ramapuram, Chennai, Tamilnadu, India; 3 Department of Prosthodontics, Crown and Bridge and Implants, Yamuna Institute of Dental Sciences and Research, Yamunanagar, Haryana, India; 4 Interna, Kalinga Institute of Dental Sciences, KIIT Deemed to be University, Patia, Bhubaneswar-751024, Odisha, India; 5 Department of Conservative Dentistry and Endodontics, Rayat Bahra Dental College and Hospital, Mohali, Punjab, India; 6 Department of Conservative Dental Sciences, College of Dentistry, Qassim University, Buraidah, Saudi Arabia

**Keywords:** Color stability, composite, surface roughness

## Abstract

Composite resins can get discolored for a variety of reasons, both internal and external. Therefore, it is of interest to assess a composite resin's surface roughness and color stability following exposure to artificial saliva, orange juice, tea and Coca-Cola on days six, twelve and twenty-four. Based on beverages, 48 composite resin disc specimens were randomly assigned to four equal groups: Group 1-artificial saliva- control, Group 2-Coca-Cola, Group 3-tea and Group 4-orange juice. Surface roughness (Ra) and color change (ΔE) baseline readings were collected using a profilometer and digital image analysis technique in the CIE l*a*b scale. The readings (ΔE) were then obtained on the sixth, twelfth and twenty-fourth days after exposure to the corresponding beverages. The Coca-Cola group had the highest surface roughness (Ra) value and color variations, followed by orange juice and tea. The control group (artificial saliva) had the lowest surface roughness value.

## Background:

Patients are frequently motivated to seek dental care since aesthetics are a modern necessity. Increased strength, better management and enhanced polishability for superior optical properties are the results of the ongoing development and development in the field of aesthetic restorative materials. The idea of aesthetic dentistry has been completely transformed by composite resins. The surface smoothness of resin composites is directly influenced by the sizes and shapes of filler particles, the coupling agent and the resin matrix structures [1]. Gloss, surface roughness and color stability are the main factors that affect the restoration's appearance when using composite resin materials for direct tooth-colored restorative reasons [2]. Composite resins can get discolored for a variety of reasons, both internal and external. Permanent internally induced discolorations are caused by the type and quantity of filler, the grade of the polymer and the synergist that is included in photoinitiator system. The resin's conversion rate and physicochemical properties, particularly the water sorption rate, influence its affinity for extrinsic stains. The surface or subsurface of the resin restorations in the oral cavity may become discolored due to superficial degradation or a minor penetration and adsorption of staining agents at the superficial layer of the composite resins [3]. The consequence of different sealants, bleaching agents, polishing agents and juices on the surface roughness and color stability of nanohybrid composites have been extensively studied in the literature, but tea, coffee and Coke-all of which are frequently consumed beverages-have not [1]. In India, the majority of people drink tea and coffee. The younger generation consumes more aerated drinks like Coca-Cola. These may be more prone to color change because of the coloring chemical they contain, as well as surface deterioration of composite resin because of their mild acidity [4]. A restoration's clinical performance in the oral environment is significantly influenced by its surface quality or texture.

The arithmetic average height of roughness component abnormalities from the mean line measured inside the sampling length is the surface roughness value, represented by de Moraes [5]. In order to create a customized smile, the most crucial features of aesthetic restorative materials are color stability and surface texture. The preservation of color over the course of a restoration's functional life is crucial to the treatment's long evity [6]. Both internal and external factors influence the color stability of resin-based composites. Chemical changes in the material, such as the hydrolysis of the interface between the filler particles and the polymer matrix and the oxidation of unreacted monomers and photo- initiator components that are not consumed after exposure to light, are what produce intrinsic discolouration. Diffusions of ions and colors that can build up on the composite resin along with water are what cause extrinsic discoloration [7]. Gingival inflammation, surface discolouration and secondary caries are caused by a restoration's intrinsic features, including its size, composition, distribution and kind of organic matrix. The quality, color and functionality of restorative materials in the oral cavity are significantly influenced by their surface roughness, since increased roughness reduces the restorations' durability and aesthetic appeal due to plaque accumulation [8]. To reduce treatment time and ease the clinician's task of matching colors, a recently developed monochrome RBC [omnichroma; (OM), Tokuyama Dental, Tokyo, Japan] was introduced [6]. According to research, the probability of discoloration is directly correlated with the degree of roughness on a composite resin surface [Alex]. Therefore, it is of interest to assess and compare the surface roughness and color stability of a composite resin following exposure to fake saliva, orange juice, tea and Coca-Cola on days six, twelve and twenty-four.

## Materials and Methods:

The department of conservative dentistry and endodontics conducted this *in vitro* investigation. The study employed a supra-nano spherical resin-based composite (Omnichroma Tokuyama Dental, Tokyo, Japan). UDMA, TEGDMA, mequinol, dibutyl hydroxyl toluene and UV absorber make up the material's makeup. A total of 48 samples were created by filling separate, specially designed plastic molds with composite resin that measured roughly 5.0 mm by 2.0 mm. As directed by the manufacturer, all of the specimens were light-cured using Light Emitting Diode (LED) light. For a full day, the samples were kept in a jar filled with distilled water. Four sets of twelve samples each were created from the 48 produced samples. Group 2: Coca-Cola (used in accordance with manufacturer's directions), Group 1: Artificial saliva (control group) Group 4: freshly made orange juice and Group 3: tea (made by adding 4 g of tea leaves to 300 ml of boiling water and boiling the mixture for 5 minutes). The 6th, 12th and 24th days of exposure were used to further segregate each group. Each sample's baseline surface roughness and color values were determined using a three-dimensional (3D) optical profilometer and digital imaging method, respectively. Each sample was submerged in its corresponding beverage and fake saliva for a total of five minutes, twice a day, for a total of twenty-four days. Every day, the same amount of beverages were created and restocked in order to maintain consistency. On the sixth, twelfth and twenty-fourth days of exposure, the surface roughness values (Ra) and color stability value (ΔE) were measured. The specimen's whole surface was scanned by the stylus and each sample's average surface roughness (Ra) values were calculated. A profilometer with a cutoff of 0.8 mm and a speed of 0.25 mm/s was used to record the Ra value (μm). An average was computed after three measurements were taken. Using a digital image analysis technique, the specimens' color stability was evaluated in the Commission International eI' Eclairege L*a*b*(CIELAB) color space. SPSS (version 24.0; SPSS Inc., Chicago, IL, USA) was used to analyze the collected data. Analysis at P <0.05 was done using the paired t-test, post hoc Tukey test and one-way analysis of variance (one-way ANOVA).

## Results:

Of the four groups examined, samples exposed to Coca-Cola had the highest surface roughness (Ra) value (Table 1) and color alterations (Table 2), followed by those treated to tea and orange juice. The control group (artificial saliva) showed the least amount of surface roughness and color alterations.

## Discussion:

Resin-based composite materials are widely used in dentistry as direct tooth-colored restorative treatments [9]. Composite materials may exhibit color changes as a result of frequent contact with colored foods consumed as part of a diet. Coffee, a dark solution, builds up in the restorative material's structure, decreasing its ability to transmit light [7]. To replace a range of shades, a single-shade resin composite-also referred to as a universal shade-was created. The nanofillers (nanomers) and nanomer groups (nanoclusters) in this composite resin are reported to provide improved color harmony with tooth tissues. By using a single shade that has a chameleon or blending effect, these composite resins provide shade matching for all tooth hues, enabling the composite to match the color of its neighboring teeth. One such monochromatic resin composite [2] that was utilized in this investigation is omnichroma. A number of complex processes take place when a composite restoration is inserted into the mouth, eventually leading to the restoration's deterioration. The most frequent cause of damage is reportedly toothbrush abrasion [9]. The popular soft drink Coca-Cola has the lowest pH (2.7) of all the drinks included in this study. Low pH in acidic foods and beverages has been shown to cause erosive wear on materials. High acidity may have a stronger softening impact on the resin matrix, encouraging filler particle dislodgement and leaching out and raising the composite resin's surface roughness [10]. Surface roughness has long been measured in lab studies using surface profilometers. Profilometers come in two varieties: contact profilometers and non-contact profilometers. There are two additional categories for the contact profilometer: manual and digital. A diamond-tipped stylus on the contact profilometer is used to run over a material that could harm the sample surface. One noncontact profilometer employed in the investigation is the universal 3D profilometer. It can execute several methods on the same tester. White light interferometer, spinning disk confocal microscopy, dark-field microscopy and bright-field microscopy are its four imaging modes. Combining various methods based on how they are applied on a single platform facilitates thorough data analysis and lowers maintenance costs [11, 12]. Composite resin discoloration frequently necessitates their replacement because it detracts from the restoration's aesthetic result. The outcome of an aesthetic repair is therefore significantly influenced by the resin composite's color stability [13]. Both visual and instrumental methods can be used to evaluate the color of composite resins. Although utilizing shade guides to visually judge color is a popular practice, it is subjective, inconsistent and may differ from observer to observer. However, a more objective, quantitative, accurate and repeatable color evaluation is made possible by instrumental color assessment using spectrophotometers, colorimeters and digital image analysis [14]. Yellow colorants of various polarities are found in tea. First to elute are components with higher polarity, such as those found in tea. Coca-Cola generated a small hue shift in the current investigation, which may have been caused by the samples' altered roughness as a result of the solution's low pH (2.62). Because caramel color is present in its composition, cola acquires its hue. These findings support the claim made by Patel *et al.* [15] that Coca-Cola only slightly alters the color of composite resins. In 2005, Jarad *et al.* determined that the digital camera may be utilized for color measurements in the dental clinic after finding a very high and statistically significant correlation between the spectrophotometer and digital camera for all CIE L*, a* and b* color coordinates [16]. In every examined time period, surface roughness and color change both showed time-dependent increases; the lowest values were recorded on the sixth day, followed by the twelfth day and the highest value was recorded on the twenty-fourth day. These findings are consistent with research conducted by Chandrasekhar *et al.* [17]. Following exposure to coffee, Coca-Cola, tea andartificial saliva on days seven, fourteen and twenty-eight, Chowdhury *et al.* assessed the surface roughness and color stability of a nanohybrid composite resin. They came to the conclusion that both surface roughness and color change increased over time [1]. The outcomes align with our conclusions. Following toothbrush simulation and immersion in coffee and an aerated beverage, Alex and Venkatesh assessed the surface roughness and color stability of a single shade and multi-shade composite resin. They came to the conclusion that single-share composite resins are more likely than multi-shade composite resins to cause discolouration in beverages [9]. Following exposure to a desensitizing agent, Gupta *et al.* assessed the surface roughness and color stability of four distinct composite resin restoration types. They came to the conclusion that after being exposed to the desensitizing agent, nanofilled composite resin restorative material had very little surface roughness and color change [18]. Gürses *et al.* examined the color stability of various universal composites after immersing them in water for a week prior to coloring. They came to the conclusion that storing the samples in distilled water for a week before to staining did not lessen the samples' color change values [19]. Using smart chromatic technology, Cumhur and Özkoçak evaluated the color stability and surface roughness of a newly created single-shade resin-based composite (RBC) against nanohybrid and nanoceramic RBCs. They came to the conclusion that because the new generation monochromatic smart composite resin with spherical filler (OM) exhibits acceptable values in terms of color stability and roughness, it is clinically recommended. Because OM makes color selection easier, it might be a better option than multi-shade composites [6]. Significant variations in the color stability of ceramic materials upon exposure to staining solutions were discovered by Patil *et al.* They stated that, Zirconia demonstrated exceptional stain resistance [20]. Surface roughness and color changes in artificial saliva, tea, orange and Coca-Cola were found to be positively correlated in the current investigation. Color change increased together with surface roughness, suggesting a decrease in the composite resin's color stability.

## Limitations:

Both parameters were assessed for up to 24 days in the current investigation; however, more long-term clinical trials or *in vitro* studies with improved oral environment simulation are required to validate the results.

## Conclusion:

Composite resin was vulnerable to surface roughness and color change when exposed to beverages within the study's constraints. Coca-Cola had the roughest surface, followed by tea and coffee. Surface roughness and color stability were both time-dependent, meaning that as time increased, so did surface roughness and color change.

## Figures and Tables

**Table 1:** Surface roughness of all groups after exposure to beverages

**Groups**	**Mean**	**df**	**F**	**p**
Group I- control	0.019	30	159.353	0.01
Group II- coca cola	3.874	30	32,026.65	0.01
Group III- Tea	5.48	30	25,521.26	0.01
Group IV- orange juice	10.745	30	82452.643	0.01
df: Degrees of freedom,
F statistics;
P-Probability value,
test used-ANOVA test

**Table 2:** Color change of all groups after exposure to beverages

**Groups**	**Mean**	**df**	**F**	**p**
Group I- control	0.024	25	1745.643	0.01
Group II- coca cola	60.53	25	3064.54	0.01
Group III- Tea	19.154	25	13,501.64	0.01
Group IV- orange juice	17.432	25	20,063.54	0.01
